# Analysis of Misdiagnosis of 4 Cases of Tuberculosis of Thyroid and Literature Review

**DOI:** 10.1155/2012/862595

**Published:** 2012-12-27

**Authors:** De-Tao Yin, Wenxun Wu, Shengli Cao, Hongqiang Li

**Affiliations:** ^1^Department of Thyroid Surgery, First Affiliated Hospital of Zhengzhou University, Zhengzhou, Henan 450052, China; ^2^Key Discipline Laboratory of Clinical Medicine, Zhengzhou, Henan 450052, China; ^3^Department of Endocrinology, First Affiliated Hospital of Zhengzhou University, Zhengzhou, Henan 450052, China

## Abstract

*Objective*. To investigate the cause of misdiagnosis and the diagnosis and treatment of tuberculosis of thyroid. *Methods*. Four cases of tuberculosis of thyroid were reported and the related literature was reviewed, as well as the causes of misdiagnosis and the diagnosis and treatment were discussed. *Results*. Two cases were misdiagnosis as thyroid adenoma and one case as thyroid carcinoma, while one case as missed diagnosis. Part of resection, local excision, and lobectomy was performed, respectively, with all the patients who were treated with antituberculosis drugs and recovered. *Conclusion*. The atypical manifestation of tuberculosis of thyroid suggested that it is important to reinforce the knowledge of this disease. Cytological examination by fine-needle aspirate biopsy was helpful to the diagnosis. The first choice was treatment with anti-tuberculosis drugs.

## 1. Introduction

Tuberculosis (TB) of thyroid was also called tuberculous thyroiditis. Tuberculosis had been described in many parts of the body, but the involvement of the thyroid gland was rare occurrence. The diagnosis of tuberculosis of the thyroid was not easy because there were not any specific symptoms to show the clinical feature of tuberculosis [1–3]. The following analyses of misdiagnosis of 4 cases of tuberculosis of thyroid were from department of thyroid surgery, the first affiliated hospital of Zhengzhou university, Zhengzhou, China, during June 2002 to April 2007.

## 2. Case Report 

### 2.1. Case 1

A 20-year-old woman was hospitalized with one-month history of thyromegaly. The patient was a nurse and worked in a tuberculosis hospital for 2 years. She denied suffering from TB before. Neck examination revealed a nontender and firm nodule which was in levels II goiter, measuring 2.0 cm × 1.5 cm. The nodule moved with deglutition. A chest X-ray and thyroid function tests were normal. The blood count was as follows: HB 110 g/L and RBC 3.78 × 10^12^/L. Erythrocyte sedimentation rate (ESR) was 25 mm/h. A thyroid ultrasonography was performed which showed heterogeneity and the presence of a hypoechogenic nodule on the lower part of the left lobe. Thyroid radionuclide scanning showed the lower pole of left lobe was “cold nodule.” Admitting diagnosis was left thyroid adenoma. The surgical finding was a 2.5 cm × 2.0 cm × 2.0 cm nodule and part of resection was performed on the left thyroid gland. The section of tumor was grayish red. There was cheese-like substance. Both the frozen section and pathological examination showed thyroid tuberculosis. Anti-TB chemotherapy was commenced after the operation. The patient remained asymptomatic in 9-year followup.

### 2.2. Case 2

A 34-year-old woman was admitted to our hospital with a 2-day history of a right neck mass, which was not accompanied with inertia, low grade fever, and night sweating. Neck examination revealed a 1.5 cm × 1.0 cm nontender and firm nodule with ill-defined margins, which was in levels I goiter. A chest X-ray and thyroid function tests were normal. Ultrasound examination of the thyroid revealed an enlarged 2.0 cm × 2.0 cm × 2.0 cm cystic mass in the lower pole of the right lobe with internal echoes. Thyroid radionuclide scanning showed the lower pole of right lobe was “cold nodule.” Admitting diagnosis was right thyroid cystadenoma. The surgical finding was right thyroid cystadenoma, whose surrounding was adhesion heavily. When the adhesion was ruptured, it was gone out of off-white, thin pus. Culture was positive for mycobacterium tuberculosis. The diagnosis of thyroid TB was made then. Lesion debridement was done and local was irrigated with streptomycin. The routine pathology of abscess wall showed tuberculosis of thyroid. Anti-TB chemotherapy was commenced after the operation. The patient remained asymptomatic in 7-year followup.

### 2.3. Case 3

A 68-year-old woman was admitted to our hospital with a 20-year history of neck masses, which become aggravate for 2 months. Two months ago, the neck masses became larger because of fever and flu. Physical examination showed weight loss, appearance of anemia, and the temperature 38°C. Neck examination revealed both thyroids were in levels II with a mass in right lobe of thyroid gland which reached approximately 3.5 cm × 3.0 cm with hard surface, no tenderness, and ill-defined margins. The left side of the surface was not smooth. Several swollen lymph nodes were around right carotid artery, and one fusion was of about 2.0 cm × 2.0 cm × 1.5 cm. The blood count was as follows: HB 105 g/L and RBC 3.65 × 10^12^/L. Erythrocyte sedimentation rate (ESR) was 30 mm/h. A chest X-ray and thyroid function tests were normal. A thyroid ultrasonography was performed which showed the volume of bilateral thyroid lobe was significantly increased with abnormal shape. The right middle lobe may be a substantial exploration and low echo-ray missions with unclear boundaries which was seen in the hyperechoic spot. The left lobe could be explored by a number of mixed clumps and heterogeneous echo texture. Thyroid radionuclide scanning showed the right lobe of the central was cold nodule. Spiral CT of neck showed the substantive of the right central thyroid with neck lymph nodes intumescence. It suggested that it should be the right side of thyroid cancer with lymph node metastasis. Admitting diagnosis was right thyroid carcinoma with lymph node metastasis. The surgical finding was that the right middle thyroid solid mass was about 3.5 cm × 3.0 cm × 3.0 cm, and adhesion between the substrate and the surrounding was heavy. The left thyroid multiplied cystic masses and the enlargement of lymph nodes were around the right neck vessels. A subtotal of resection of the left thyroid gland, resection of the isthmus, and lobectomy of the right thyroid gland were performed. Quick frozen and conventional pathologic findings were the right tuberculosis of thyroid with the left nodular goiter. Anti-TB chemotherapy was commenced after the operation. The patient remained asymptomatic in 6-year followup.

### 2.4. Case 4

 A 28-year-old woman was admitted to our hospital with a history of neck masses, which were accompaniment with exophthalmos, emaciated, fatigue, intermittent palpitation, no cough, fever, night sweats, and other symptoms. On several occasions the consultation was as primary hyperthyroidism in other hospitals. The patient was conducted regular internal medicine treatment, but the efficiency was poor. Physical examination revealed weight loss, HR 110 times/min, moderate infiltrative exophthalmos, and III degree of bilateral thyroid enlargement. A mass approximately 1.5 cm × 1.0 cm with smooth surface was palpable on the right inferior thyroid pole. Thyroid ultrasonography showed bilateral thyroid diffuse increased with rich blood supply and the right lower pole could explore a substantial low-light echo groups with the border clearly seen in the patchy echogenic echoes which suggested that it was hyperthyroidism with the right side of the thyroid placeholder. Thyroid function tests were preformed: serum thyroid stimulating hormone TSH = 0.01 mU/mL, FT4 = 153.5 pmol/L, and FT3 = 35.5 pmol/L. A chest X-ray showed old tuberculosis in the lower lobe of the right lung. Admission diagnosis showed primary hyperthyroidism with thyroid adenoma on the right. The surgical finding was bilateral thyroid diffuse increased with rich blood supply and the right lower pole could explore a 1.5 cm × 1.5 cm × 1.0 cm nodule and bilateral subtotal thyroid resection was performed. The section of tumor was grayish red and there was cheese-like substance. Histopathology revealed toxic goiter with right lower pole of thyroid TB. Anti-TB chemotherapy was commenced after the operation. The patient remained asymptomatic in 5-year followup.

## 3. Discussion

 Although tuberculosis has been reported in many parts of the human body, thyroid involvement is extremely rare. The exact reason for the rarity of the tuberculosis of thyroid is unknown. However, there were several possible explanations: (1) the thyroid gland is made up of colloid material possessing bactericidal action; (2) blood flow to the thyroid is extremely high, and the gland stores an excess of iodine; (3) destruction of tubercle bacilli may be enhanced by increased physiological activity of phagocytes in hyperthyroidism; (4) thyroid hormones exercise anti-TB roles [4, 5]. Thyroid tuberculosis was recognized with a rate of 0.4%–1% in foreign countries, while the rate was 0.4%–0.76% in China [1, 2]. 

Solitary thyroid nodule was the main performance for thyroid TB, while some as solid or cystic nodules. Infiltrating the adjacent tissue could cause dyspnea, dysphagia, hoarseness, and so on. Because there was no specific symptom, thyroid TB was often misdiagnosed as thyroid adenoma or thyroid carcinoma. The diagnosis was based on the presence of epithelial cell granulomas with peripheral lymphocytic cuffing, Langhans giant cells and central caseation necrosis [6–8]. Auxiliary tests revealed that fine needle aspiration cytology (FNAB) had important diagnostic value. If Langerhans cell, cheese-like substance, and interstitial cells were found, or puncture and extraction of pus, such as acid-fast stain and acid-fast bacilli were found, the diagnosis was confirmed [9, 10]. Radionuclide bone scan, iodine absorption rate measurement, CT examination, and ultrasonography could suggest thyroid placeholder, but it was not specific, and easily misdiagnosed. The pathological type classification is divided into 4 types [2]. (1) Cheese type: the most common clinical, pathological changes to caseous necrosis, are often accompanied with bleeding cystic change. Preoperative fine needle aspiration or surgery, we can see cheese-like substance or a cold abscess, thyroid abscess wall tissue fibrosis, with the surrounding tissue adhesion, and compression of the trachea, esophagus, recurrent laryngeal nerve, and so forth, preoperative diagnosis is often misdiagnosed as thyroid adenoma, The cases of 1, 2, and 4 were found to be cases of this type. (2) Granuloma type: it is often seen. Hard texture, lesions from the proliferative form of tuberculous granuloma, which is surrounded by fibrous tissue hyperplasia, is often misdiagnosed as clinical thyroid cancer. The case of 3 is of this type. (3) Diffuse type: clinical rare. In diffuse thyroid enlargement, nodular, the surface is not smooth, clinical manifestations are similar to diffuse goiter while pathologic is similar to wood-like goiter. (4) Military type: rare. There was no significant increasing of thyroid; most of the general miliary tuberculosis in the thyroid is the partial performance with small miliary lesions.

Misdiagnosis was often made because of the reasons as follows. (1) There was a lack of knowledge of the disease. Because of its low incidence, history taking was not fine. It paid a little attention to the diagnosis and differential diagnosis, for example, case 1, patients with history of close contact with TB patients, and case 4 with the right upper chest with an old tuberculosis of the lung, but were not got enough attractiveness. (2) Clinical manifestations of this disease were not specific and laboratory techniques and laboratory examinations were in lack of specificity. (3) Satisfied with the combined diagnosis of disease, while ignoring the diagnosis of the disease: as described in case 4, this disease is coexistence with hyperthyroidism. The following points are helpful to improve the diagnosis of thyroid nodules: (1) young adults, especially women; (2) people with previous history of TB or close contact with TB history, or the existence of other parts of the TB lesions, (3) thyroid mass in the short term: there were weight loss, fever, ESR step up, and other TB symptoms; (4) thyroid nodules were located in the lower right pole of the thyroid with nearby lymph nodes while it was not significantly increased; (5) for those suspected thyroid nodule, FNAB was a simple and effective method of diagnosis. (6) if thyroid tuberculosis was highly suspected, short-term antituberculosis treatment is useful. If significant improvement happened, it might be also helpful for diagnosis. (7) if the lesions with caseous necrosis were found during surgery, rapid frozen section should be performed. 

Tuberculosis had been reported in many parts of the body [11–13]. Regardless of which type, it is regular whole-body antituberculosis treatment should be done. Because thyroid tissue was blood rich and tuberculosis activity uncertain, drugs were easy to achieve with the accumulation of systemic. Treatment of thyroid tuberculosis consists of antituberculous drugs combined with surgical removal of the affected parts of the thyroid gland or surgical drainage. Anti-TB drugs plus puncture pus into the antituberculosis drug treatment were better. It not only helped patients not to suffer from surgery but also reduced the burden of patients on the drug expense. It should be the first choice [11–14]. Treatment using rifampicin, rimifon, and ethambutol plus pyrazinamide was longer than conventional treatment and paid attention to nutrition support therapy. Although the surgical treatment of thyroid tuberculosis is effective, the surgical indications of extrapulmonary TB were significantly reduced because of the success of chemotherapy in tuberculosis treatment. In those whose diagnose are not confirmed before surgery, intraoperative frozen section examination should be performed. If it was for the granulomatous type, thyroid lesion resection or partial resection should be performed. Diffuse swelling, and the undecided nature of tracheal compression symptoms, the removal of the isthmus should be done to relieve symptoms. Type of cheese had formed a tight adhesion around the abscess and should not have lesions completely removed, so as not to damage nerves, blood vessels, possible drainage of abscess drainage, and local irrigation with antituberculosis drugs should be done. If there was secondary infection, it should be plus antibiotics [15]. The formal antituberculosis treatment should be done after the surgery. The prognosis of thyroid TB was good. After surgery, the lesions were mostly without recurrence.
